# A quest for cytosolic sequons and their functions

**DOI:** 10.1038/s41598-024-57334-1

**Published:** 2024-04-02

**Authors:** Manthan Desai, Syed Rafid Chowdhury, Bingyun Sun

**Affiliations:** 1 Department of Molecular Biology and Biochemistry, Simon Fraser University, Burnaby, BC Canada; 2 Department of Computing Science, Simon Fraser University, Burnaby, BC Canada; 3 Department of Chemistry, Simon Fraser University, Burnaby, BC V5A 1S6 Canada

**Keywords:** Sequons, Protein structure and function, Asx turns, β-turns, Posttranslational modifications, N-glycosylation, Transmembrane domains, Membrane protein topology, Zinc finger proteins, Protein-DNA binding, Biochemistry, Chemical biology

## Abstract

Evolution shapes protein sequences for their functions. Here, we studied the moonlighting functions of the N-linked sequon NXS/T, where X is not P, in human nucleocytosolic proteins. By comparing membrane and secreted proteins in which sequons are well known for N-glycosylation, we discovered that cyto-sequons can participate in nucleic acid binding, particularly in zinc finger proteins. Our global studies further discovered that sequon occurrence is largely proportional to protein length. The contribution of sequons to protein functions, including both N-glycosylation and nucleic acid binding, can be regulated through their density as well as the biased usage between NXS and NXT. In proteins where other PTMs or structural features are rich, such as phosphorylation, transmembrane ɑ-helices, and disulfide bridges, sequon occurrence is scarce. The information acquired here should help understand the relationship between protein sequence and function and assist future protein design and engineering.

## Introduction

Through evolution, protein sequences dictate protein structures and highly influence their functions. With the rapidly available proteome sequences spanning all biological species, it is possible to systematically investigate sequence effects on protein function. Here, we studied sequons, the sequence motif recognized for N-glycosylation in membrane and secreted proteins. We are particularly interested in the potential functions of sequons in cytosolic and nuclear proteins.

N-glycosylation is one of the most abundant post-translational modifications (PTMs) in biological systems and plays crucial roles in protein folding, trafficking, homeostasis, secretion, and protein–protein and protein-glycan interactions in signaling and immune responses^1,2^. As a result, malfunction of N-glycosylation can cause many devastating diseases, such as cancers, congenital disorders of glycosylation, cardiovascular, neuron degenerative, and autoimmune diseases^1,3^. Wealth information has gained in N-glycosylation to protein structure and function and particularly to membrane, secreted, and extracellular matrix proteins, because this PTM is known to be present on the ectodomain of membrane proteins^4^.

N-glycosylation modifies the asparagine residue residing in a conserved sequence motif called the N-linked sequon, i.e., a three amino acid motif of NXS/T, in which X can be any amino acid but not P. Numerous studies have focused on the properties of sequons that can be N-glycosylated, and various knowledge has been accumulated, and several algorithms are available to predict the N-glycosylation efficiency of sequons^5–8^. In most of these studies, sequons were only studied in glycoproteins.

However, sequons are ubiquitously present in protein sequences. Apiwler et al. analyzed the SwissProt database over 20 years ago^9^ and identified more than 60% of proteins containing sequons, yet not all of them were N-glycoproteins. Furthermore, in our recent study of N-glycosylation and transmembrane domains (TMs) of membrane proteins, we observed that in TM N-glycoproteins, the number of sequons conflicts with the predicted membrane protein topology, i.e., sequons reside on the cytoplasmic side of the membrane, sharply drops with single conflict/protein having the highest frequency. This observation suggests that the sequon location is not random in membrane proteins but is preferred in ectodomains.

These observations raised our interest in studying cytosolic sequons—a feature that has seldom been investigated. In particular, we wanted to address the following questions: (1) Is there a rule to guide the presence or absence of sequons in protein sequences? (2) Will there be other functions carried by sequons besides N-glycosylation?

Using the annotated human proteome from SwissProt, we discovered that a large number of nucleocytosolic proteins also carry sequons, and we named them cyto-sequon proteins. By comparing the properties of cytoproteins to those of membrane and secreted proteins, we discovered the unique properties in cyto-sequons and deduced their potential functions.

## Methods

### Sequon identification

We analyzed all reviewed human proteins in SwissProt for the presence of sequons (NXS/T, and X is not P) and, if so, their specific locations.

### Membrane protein prediction

We used Deep TMHMM^10^ based on neuron network to predict the transmembrane (TM) domain and membrane orientation of the reviewed human proteome from SwissProt. From the obtained results, we divided the total proteome proteins based on their cellular compartments into intracellular (cytoproteins), membrane (TM proteins), and extracellular (secreted proteins) proteomes.

### Classification

Using the sequon and the abovementioned three cellular compartments, we classified the sequon-bearing human proteome as cyto-sequon proteins, TM-sequon proteins, and secreted-sequon proteins. The non-sequon proteins were named cyto-sequon free proteins, TM-sequon free proteins, and secreted-sequon free proteins. In TM-sequon proteins, based on their TM topology, we further classified sequons that were localized in the ectodomains as ecto-sequons and those localized in the cytoplasmic domains as endo-sequons.

### Protein length and sequon density analysis

For each protein class, the distributions of sequons and protein length were analyzed. The correlations among sequon density and normalized density (i.e. sequons/protein and sequons/500 aa), spacing (i.e. fragment length/sequon), and protein length were further studied.

A linear model was built based on the protein length and sequon frequency, and least square linear regression was carried out for all sequon-bearing protein classes. The predicted intercepts at 0 sequon in the x-axis were used for the validation of the model in the sequon-free protein classes.

Quartile analyses for outliers were carried out in both sequon-free and sequon proteins. Specifically, the upper cutoff value for outliers was defined as upper quartile value plus 1.5 of interquartile range in each protein class. The interquartile range was determined by the difference between the upper and lower quartiles.

### Functional and structural analyses

To obtain molecular insights into various protein classes and subclasses we defined, functional and feature enrichment analyses were carried out by DAVID, a web server for functional enrichment analysis and functional annotation of gene lists^11^. The particular categories were examined, including the “biological processes”, “cellular localization”, and “molecular functions” in Gene Ontology, the protein domains in Interpro, and the PTM keywords and sequence features in UniProt.

### Sequence pattern analysis

To obtain hidden patterns of the selected protein domains, we used the visualization tool IceLogo^12^, developed by the Gevaert group. In particular, varied lengths of amino acid sequences around sequon or C2H2 zinc finger DNA binding domains were analyzed by IceLogo for amino acid enrichment at particular locations.

## Results and discussion

### Sequon analysis

**Figure 1:**
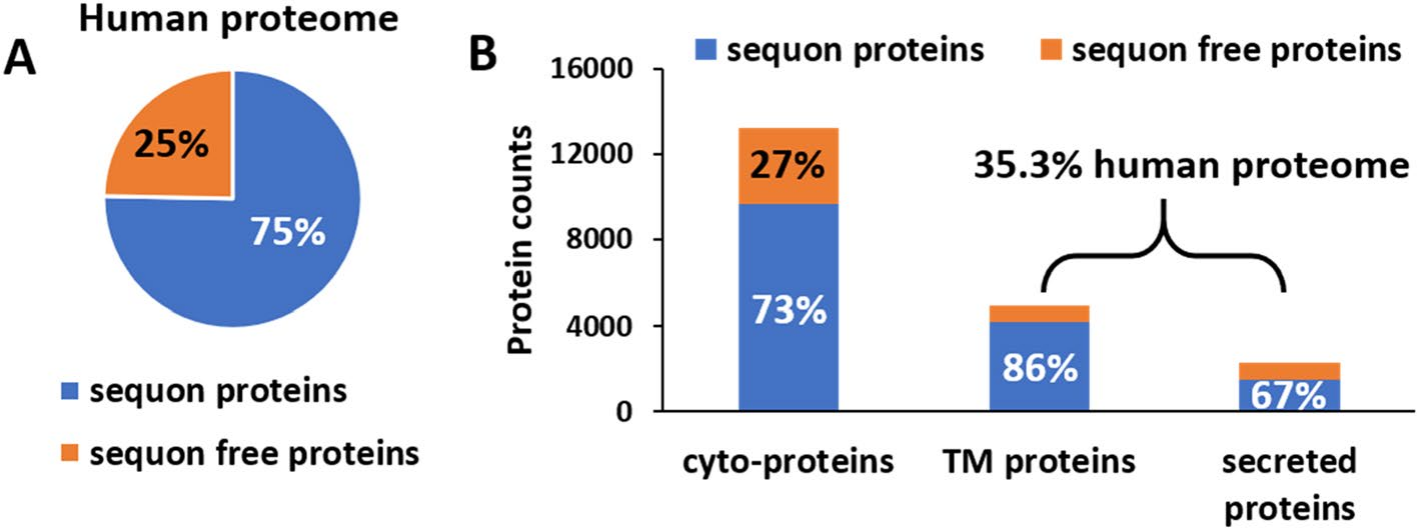
Distribution of sequon and sequon-free proteins in human proteome. (**A**) The total distribution of sequon and sequon-free proteins in human proteome; (**B**) The distribution of sequon and sequon-free proteins in cyto, transmembrane (TM), and secreted proteins.

From 20,411 SwissProt reviewed human proteins, only 25% (5040 entries) did not carry sequons, as shown in Fig. 1A. In addition, 4947 proteins were predicted by the latest Deep TMHMM^10^ algorithm to have TM domains, and 2266 proteins were predicted to be secreted. Together, the membrane and secreted proteins were only 35.3% of the human proteome. These results gave rise to 9663 (73% of total intracellular proteins) cyto-sequon bearing proteins, as shown in Fig. 1B, which was 47% of the total human proteome and 34% more than all the membrane and secreted sequon-bearing proteins combined. These were significant numbers and prompted us to further examine the potential underlying rules and functions. We named these proteins cyto-sequon proteins compared to TM-sequon and secreted-sequon proteins that are likely to be N-glycosylated. The corresponding sequon proteins in TM and secreted proteins were 4183 (86% of total TM proteins) and 1525 (67% of secreted proteins), respectively, as shown in Fig. 1B.

**Figure 2:**
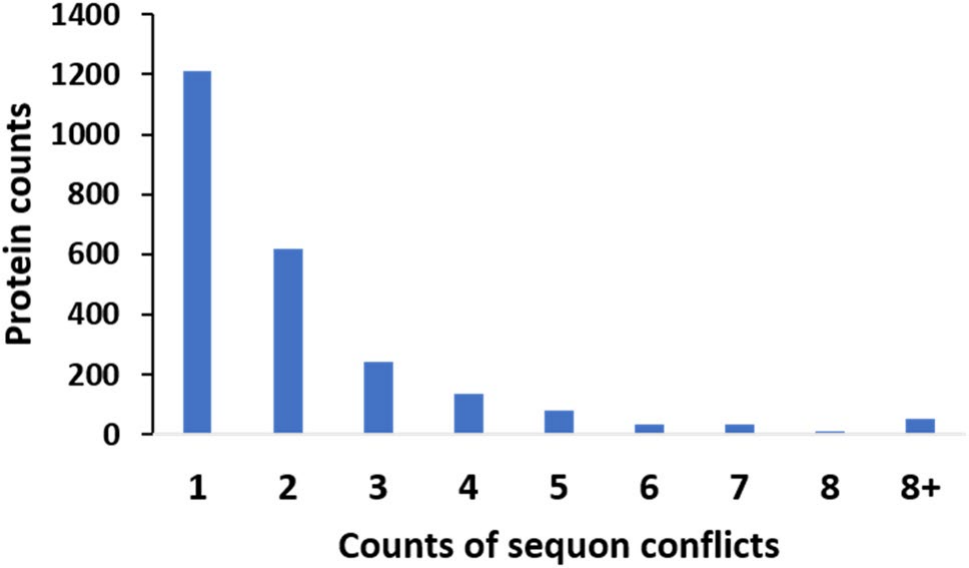
The distribution of sequon conflicts in transmembrane (TM) proteins. The conflicted sequons (endo-sequons) are ones predicted to reside in the cytoplasmic side of the membrane.

Our results were similar to a previous study on SwissProt glycoproteins^9^, in which a previous report had hypothesized that the sequons in the majority of the glycosylated proteins were utilized for glycosylation. From our recent studies analyzing the conflicts between the predicted ectodomains and the N-glycan and sequon locations in N-glycosylated TM proteins^13^, it is clear that most N-glycans in TM proteins were located in the ectodomain (i.e. ecto-sequons). Here, we further analyzed such conflict entries (i.e. endo-sequons) in all TM proteins for their sequons localizing in the intracellular domains (Supplementary Table S1). The distribution of sequon conflicts following an exponential decrease (Fig. 2), similar to those in the TM N-glycoproteins^13^.

The membrane topology is the most reliable prediction of whether a sequon will be used for N-glycosylation. N-glycosylation is among a few other structural features, such as disulfide bridges and TM domains, that can occur co-translationally^4,6^, such that it is ahead of the development of major protein conformations during folding. Consequently, few protein folds can prevent N-glycosylation except for the efficiency of OST, the enzyme complex catalyzes the en bloc addition of N-glycan to the asparagine side chain in the sequon in the ER^14^. Among the structures that can interfere with N-glycosylation, the membrane protein topology, specifically the TM domains, is one of the determining factors. Sequons partitioned to the cytoplasmic side by the TM domains cannot access the N-glycosylation machinery inside of the ER and therefore lose the opportunity to be N-glycosylated. Our conflict studies mentioned above isolated all these endo-sequon cases (Supplementary Table S1).

**Figure 3:**
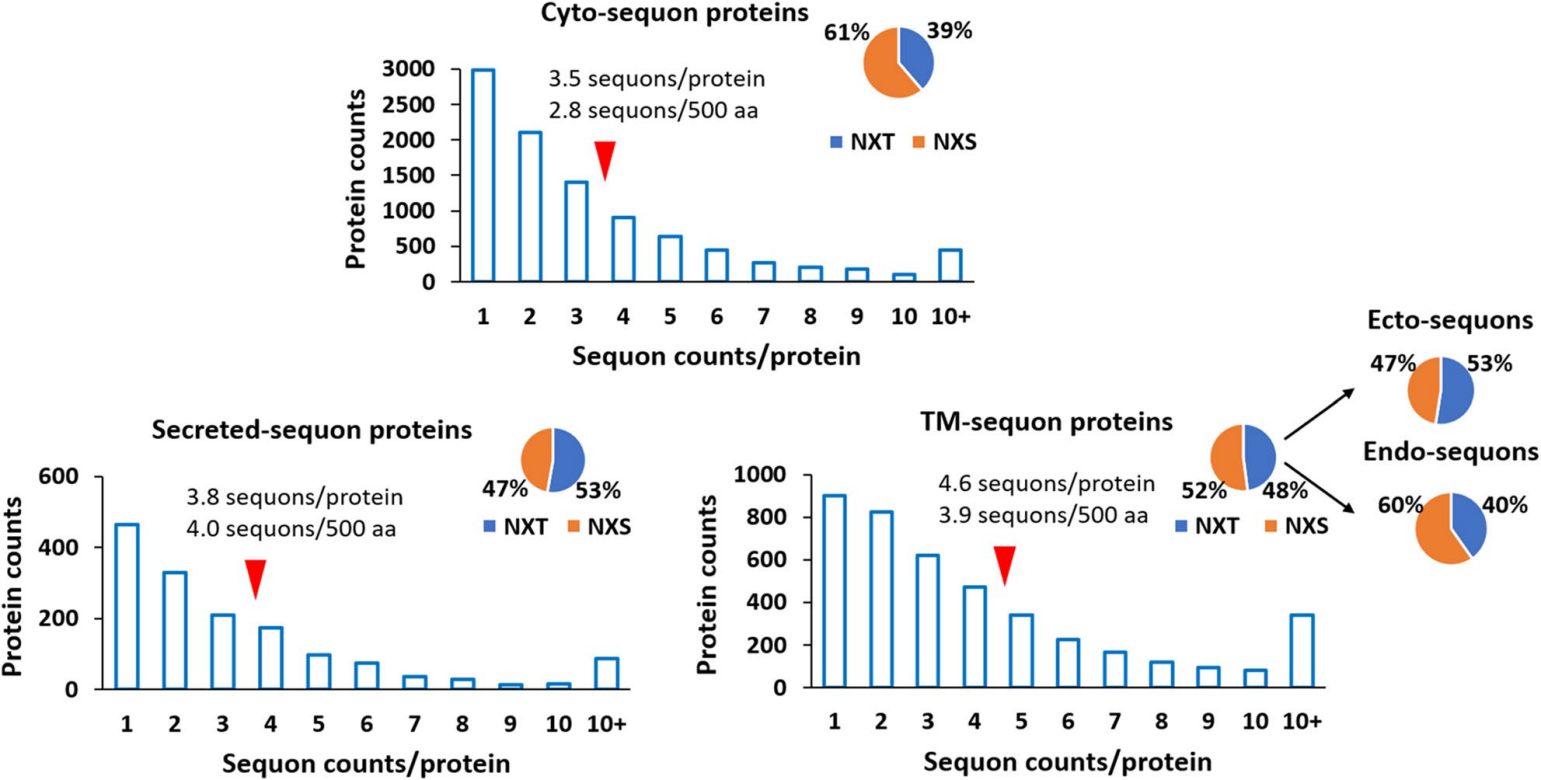
The distribution of sequons in cyto-, TM-, and secreted-proteins as a function of sequon counts/protein. The mean sequon counts/protein is also labeled by the red arrows. The normalized sequon counts/500 amino acids are displayed as well. The pie chart inserts are the distribution of NXT and NXS in cyto-, TM-, and secreted-sequon proteins and the corresponding subclasses as labeled in the figure.

Because the sequons in N-glycoproteins have experienced evolutionary selection^15–17^, we asked about the cyto-sequons. To answer this question, we first examined the S and T distribution in the sequon. Past reports have shown that due to the OST and N-glycosylation quality control systems in the ER, evolution has selected NXT over NXS in the TM and secreted proteins^15,18^. Our results agreed with these findings and showed an opposite distribution of NXS and NXT in the cyto-sequons compared to sequons in secreted proteins, as shown in the pie insert in Fig. 3. Clearly, due to the lack of the N-glycosylation machinery in the cytosol and nucleus, cyto-sequons would not experience the same Darwinian selection as ecto-sequons in TM and secreted proteins. To verify this theory, we analyzed the TM-sequon proteins. As mentioned above, our conflict analysis of TM-sequons with membrane topology divided these sequons into two subclasses: i.e. the ecto-sequons that can be utilized for N-glycosylation are therefore subjected to corresponding selection pressure, and the endo-sequons in the cytoplasmic domains that do not encounter the selection of N-glycosylation machinery. Interestingly, when we separately analyzed the NXT and NXS distribution of the ecto- and endo-sequons in the TM-sequon protein class, as shown in the insert of Fig. 3, the results agreed well with those from secreted-sequons and cyto-sequons. These results beautifully validated the previous claims in evolutionary selection^15^. However, it was unclear to us whether its opposite selection on NXS and NXT in cellular compartments reflected additional functional selections besides N-glycosylation.

To further examine the property differences among cyto-, TM- and secreted-sequon proteins, we analyzed the number of sequons carried by each protein class, and the results are summarized in Fig. 3. In general, the number of proteins decreased as a function of the sequon occurrence per protein, which was consistent across all three compared protein classes. Within the 9663 cyto-sequon proteins, the average number of sequons per protein was 3.5. The average number of sequons/TM-sequon proteins was 4.6 and that for secreted-sequon proteins was 3.8. When normalizing the sequon occurrence, i.e. sequon density, to an arbitrary 500-aa protein length unit (i.e. about the average protein length in the human proteome^15^), the values for cyto-sequon, TM-sequon and secreted-sequon proteins were 2.8, 4.9 and 3.9, respectively, as shown in Fig. 2. These results suggested that cyto-sequon proteins have the lowest sequon density among the three protein classes. It is known that amino acids carrying PTMs are more conserved in evolution due to their functional purification^19^. It is reasonable to postulate that the increased sequon density in TM and secreted-sequon proteins is because these sequons are utilized for N-glycosylation, and their presence is conserved. The none utilized sequons in cytosolic proteins can disappear over time due to genetic drift by single nucleotide polymorphisms, if they are not utilized for functional purposes^17^.

### Length and position analysis

**Figure 4:**
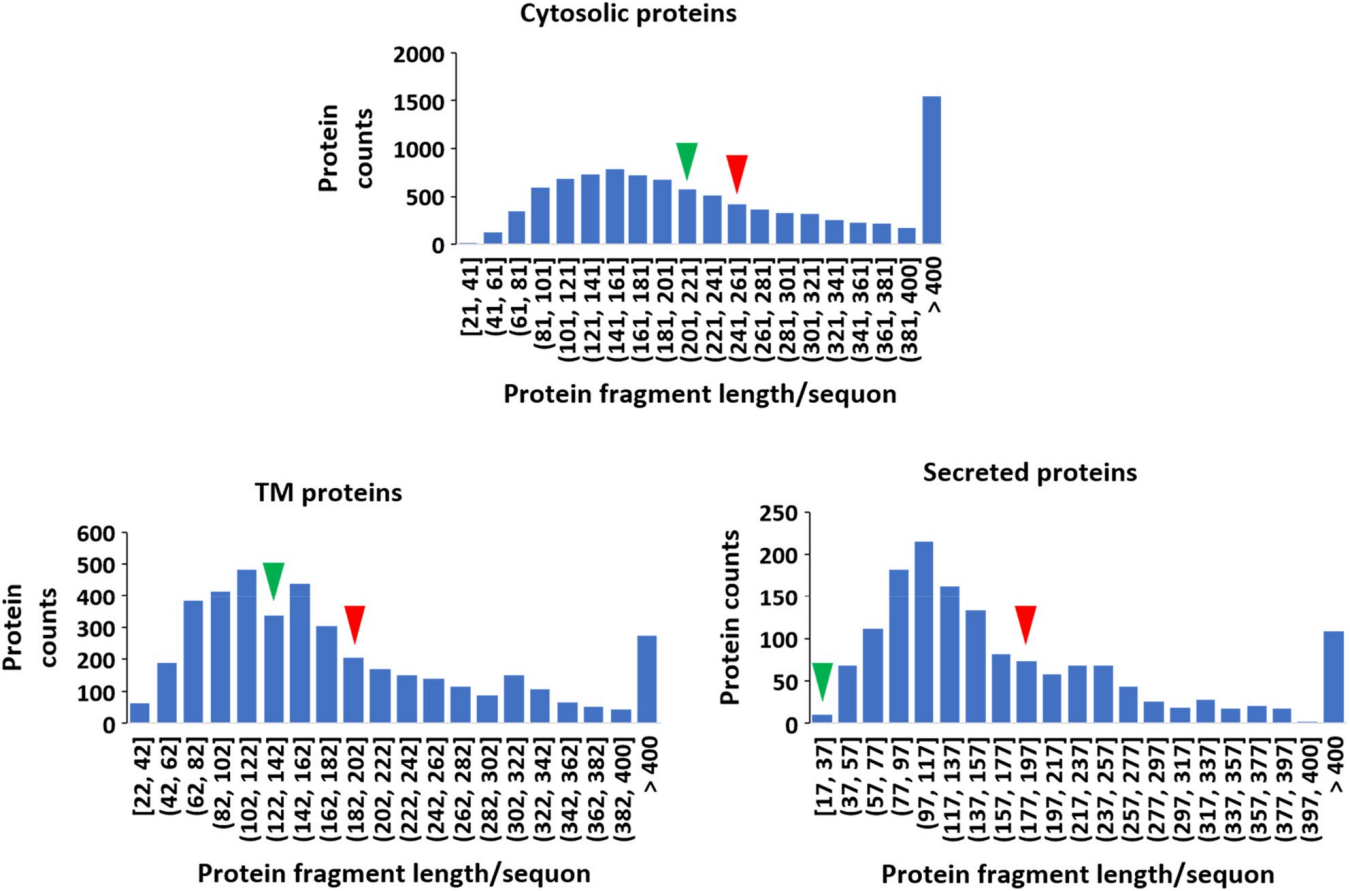
Distribution of protein fragment length/sequon in cyto-, TM-, and secreted-sequon proteins. The red arrows mark the mean value in each class, and the green arrows in each protein class marks the average fragment length for the protein in each class carrying the most sequons, i.e. Titin in cyto-sequon proteins, MUC16 (Ovarian cancer-related tumor marker CA125) in TM-sequon proteins, and Dentin sialophosphoprotein (DSPP) in secreted-sequon proteins.

The length dependence for the number of sequons in a protein, i.e. a relatively constant sequon density, has been confirmed in sequon analysis in humans as well as many other species^9,15,20^. We therefore carried out a similar analysis by studying the distribution of fragment length/sequon in a protein in each group. The results are summarized in Fig. 4. Consistent with the analysis results from the above, the cyto-sequon proteins had the longest fragment length defined by sequons (on average 259 aa/sequon), whereas the TM and secreted proteins had shorter fragments (both on average 190 aa/sequon). Clearly, the mean values indicated by the red arrows in Fig. 4 are slightly skewed to the right side of the peak distribution in the histograms shown in Fig. 4, in which the peak fragment length/sequon in cyto-sequon proteins is between 141 and 161 aa, while the values for TM proteins and secreted proteins are 102–122 aa and 97–117 aa, respectively.

Proteins carrying the highest number of sequons in each category are always interesting to introduce. For MP proteins, it is MUC16, i.e. Ovarian cancer-related tumor marker CA125, which carries 107 sequons; for secreted proteins, it is Dentin sialophosphoprotein (DSPP) carrying 67 sequons; and for nucleocytosolic proteins, it is Titin carrying 163 sequons. As the longest protein in the human proteome with 34,350 amino acids, the rich sequons discovered in Titin suggested that their presence might be caused by length. Because sequons are observed in all domains of life, their presence in proteins has been regarded as an ancient selection for stable protein folding^17^. When ranking the proteins in each group by sequon occurrence (i.e. fragment length/sequon), Titin was ranked 4996, MUC16 was ranked 1783 and Dentin sialophosphoprotein was ranked 2. Their corresponding bins in protein fragment length/sequon analysis are indicated by the green arrow in Fig. 4.

**Figure 5:**
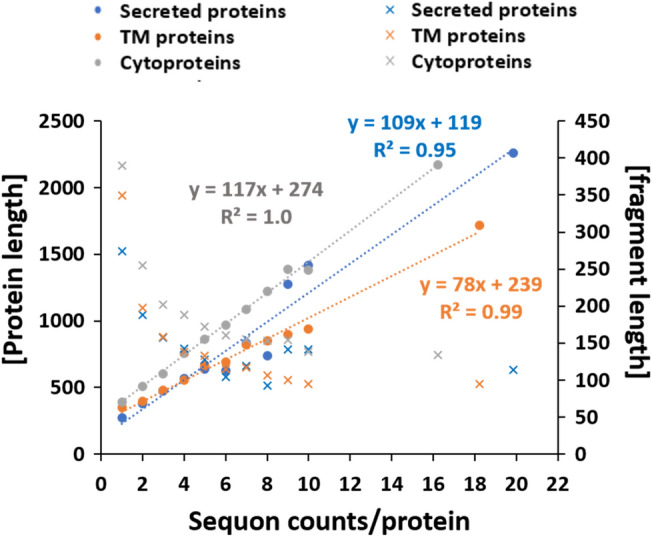
Linear regression analysis in all three sequon-protein classes of cyto-, TM-, and secreted-sequon proteins for the correlation between the protein length and sequon occurrence in one protein as the left axis (circles), and the fragment length/sequon as a function of sequon occurrence in one protein for the right axis (crosses). The fitted equations for each protein class are shown in the graph, denoted by different colors, together with their corresponding R^2^ values.

To further evaluate whether the occurrence of sequons is correlated with protein length, we analyzed the sequon counts as a function of protein length in cyto-sequon proteins and compared them with those of TM and secreted sequon proteins. The results are shown in Fig. 5. It is clear that a linear correlation exists in all three protein classes, with linear regression R^2^ values of 1.00, 0.99, and 0.95 for cyto-, TM- and secreted-sequon proteins, respectively. The cyto-sequon proteins had a higher slope of 117 than 78 and 109 for TM and secreted proteins, respectively. In addition, the intercepts for cyto-, TM- and secreted-sequon proteins were approximately 275, 240, and 110, respectively. These values suggested that proteins with lengths shorter than 275 aa, 240 aa, and 110 aa in cyto-, TM-, and secreted-sequon proteins, respectively, were unlikely to carry sequons predicted by this linear model.

**Figure 6:**
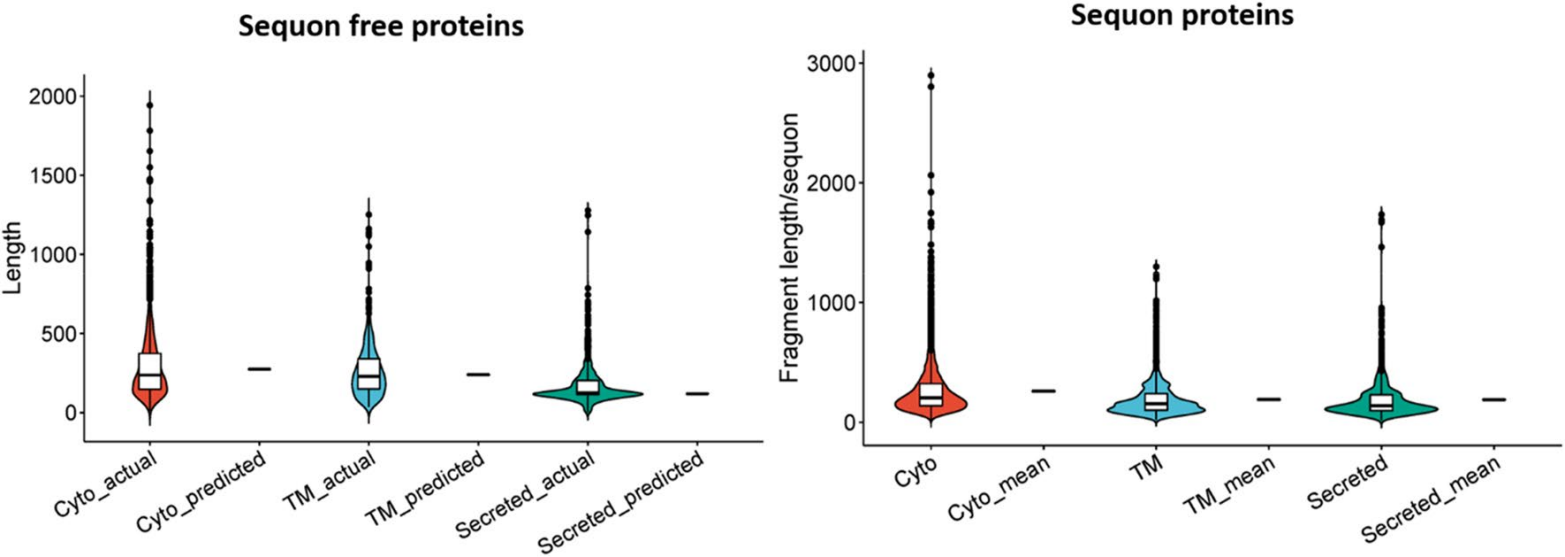
The comparison between linear model predicted and actual mean amino acid length for sequon-free proteins in all three protein classes, i.e. cyto-, TM-, and secreted proteins.

To verify the linear model prediction, we analyzed the mean length of the sequon-free proteins in all three classes, and their values were 274 aa, 262 aa and 185 aa in the cyto-, TM- and secreted-sequon-free proteins, respectively. The boxplot shown in Fig. 6 summarizes the comparison between the model prediction and the obtained actual length distribution in the corresponding protein groups. These results suggested that sequon occurrence in proteins is mainly determined by protein length; this rule applies to TM and secreted proteins as well as cytoproteins.

Interestingly, the violin plot shown in Fig. 6 indicated the existence of a large number of outliers in the upper region (Supplementary Table S2) of all protein classes. Using the quartile analysis, we defined the outlier cutoff values of 712 aa, 627 aa and 336 aa, for cyto-, TM- and secreted-sequon free proteins, respectively. The number of outliers passing the cutoff for cyto-, TM- and secreted-sequon free proteins were 129, 22, 74, respectively. Among them, the longest proteins without a sequon in the three protein groups were trichohyalin (1943 aa) in cytoproteins, cyclic nucleotide-gated cation channel 4 (CNG-4) (1251 aa) in TM proteins, and neuroblastoma breakpoint family member 20 (5207 aa) in secreted proteins.

To further understand these outlier proteins, we analyzed their Gene Ontology terms (Supplementary Table S2). The outliers in the sequon-free cytoproteins were rich of protein kinases, such as cyclin-dependent kinases and mitogen-activated protein kinase kinase kinases, and nucleoplasm proteins, including androgen receptor, OGT, and cullin 1. The outliers in secreted proteins were rich of extracellular matrix proteins, such as collagens, nidogen 1, and elastin; carrier proteins, such as apolipoproteins and albumin; proteoglycans, such as SPOCKs; and enzymes, such as matrix metallopeptidases, pepsinogens, chitinases, and carboxypeptidases.

Similar outlier analysis was carried out for the sequon bearing proteins as well using the fragment length/sequon distribution. In total, 515, 209, and 92 proteins (Supplementary Table S3) in cyto-, TM- and secreted-sequon proteins had surpassed their corresponding cutoff values of 600.2 aa, 447.5 aa, and 426.3 aa, respectively. The longest fragment/sequon in each protein group was as follows: protein AHNAK2, 5792 aa with 2 sequons (2897.5 aa/sequon) in cytoproteins; phospholipid-transporting ATPase IK (EC 7.6.2.1), 1300 aa with a single sequon, in MP proteins; and collagen alpha-2(XI) chain, 1736 aa with a single sequon, in secreted proteins. Further GO annotation discovered that for cytoproteins, intracellular signal transduction molecules were rich, including Rho- and Ras-related molecules and ATP binding proteins such as helicases, kinases, and kinesin family proteins. For MP protein outliers, the GO annotation resolved cytochrome P450 family and solute carrier family proteins. For the secreted outlier proteins, the frequent GO terms were focused on extracellular matrix, including collagens, matrilin 2, nephronectin, periostin, and proteoglycans such as agrin and tenascin. Secreted proteins such as fibrinogens, plasminogen, complement family, and enzymes such as serine protenases and matrix metallopryptidases.

If initially the presence of sequons in the protein sequences was due to their length, the outliers identified by our length linear model imply a negative selection against sequons in a subset of long proteins or long protein fragments. A further analysis of the outliers with long sequon-free protein fragments showed that the majority of these proteins are single-sequon-bearing proteins, as shown in Fig. 5, the secondary Y-axis. The percentages of single-sequon-bearing proteins in the outliers of the three protein classes were 84%, 89%, and 72% for cyto-, TM-, and secreted-sequence proteins, respectively. The dominance of single-sequon proteins in the fragment outliers suggested that these proteins might be closely related to the sequon-free outliers. We therefore combined these two subclasses as the sequon-poor class for further analysis below.

In contrast to these sequon-poor proteins, we also examined cyto-sequon proteins with super-rich sequons. Because the linear model on cyto-sequon proteins predicted a slope of 117 aa / sequon, we used a 100 aa-cutoff in fragment length/sequon to filter the cyto-sequon proteins. This resolved 1059 proteins that were considered sequon-rich cytoproteins (Supplementary Table S4).

### Functional and structural analyses

**Figure 7:**
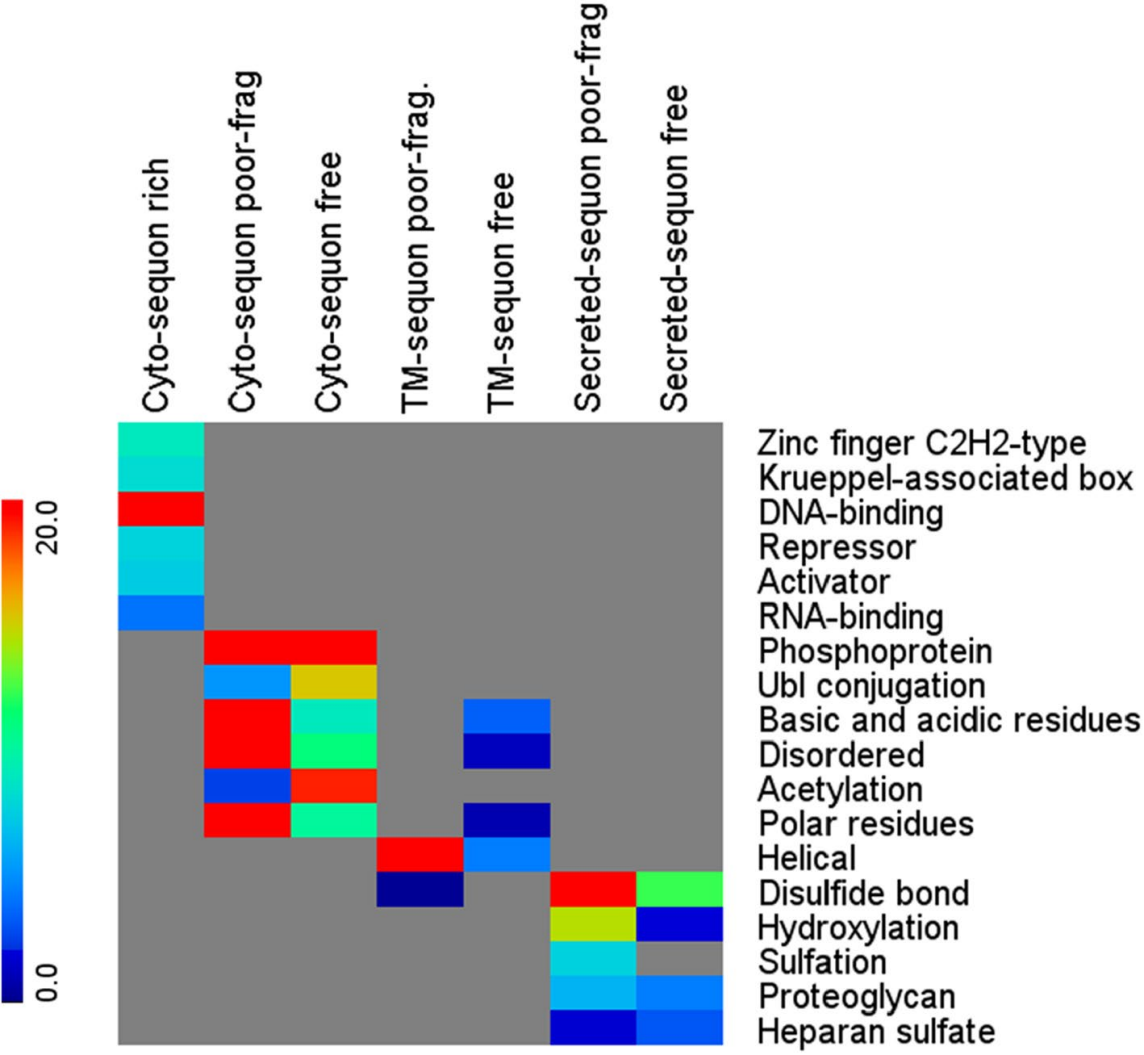
Heatmap of the enrichment analysis of UniProt features and PTM keywords in sequon-rich cytoprotein class and sequon-poor classes by DAVID, in which the sequon-poor classes are further divided by sequon-free and with long sequon-free fragment (sequon-poor frag.) The UniProt features or PTM keywords are listed in rows, and the comparing protein classes are in columns. The color encodes the -log10 transformation of the Benjamini score in DAVID. All the enriched terms were filtered by the ease score, an adjusted Fisher Exact *p*-value, of 0.1.

To understand the hidden biological functions and structural features in sequon-poor and sequon-rich protein classes, we performed a set of enrichment analyses in Gene Ontology as well as in UniProt PTM Keywords, structural features, and Interpro domains using DAVID^11^. Figure 7 summarizes the results in a heatmap, in which the color encodes the −log10 transformation of Benjamini score, which was after the adjustment of the enrichment p value in multiple testing^21^ offered by DAVID. The p-value in DAVID was computed by the Fisher Exact p-value^11^. The enrichment results were similar between the two sequon-poor classes, i.e., the sequon-free proteins and sequon-sparse proteins. The most insightful results were from UniProt PTM Keywords and UniProt structural feature analyses. In particular, the secreted sequon-poor protein class was enriched with disulfide bridges, O-glycosylation, and sulfation, and the TM protein class was enriched with ɑ-helices. In cyto-sequon-poor proteins, enrichment resolved phosphorylation and ubiquitination in PTMs and acidic, basic, polar residues and disordered regions in the corresponding structural features. The most drastic difference between sequon-free and long-sequon spacing in the cyto-sequon-poor class was the high enrichment in the sequon-free class of alkylation, such as acetylation.

These results demonstrated that the sequon-poor regions of a protein are occupied by other PTMs, which agreed with our previous studies^13^ in which we observed a complementarity between N-glycosylation, ɑ-helix TM domains, and disulfide bridges, suggesting the generalization of this rule. Such observations may be resolved from steric and spatial constraints in protein structure, in which the poor presentation of one type of PTM often gives space to the other PTMs. It could also be explained as the presence of other PTMs relieved the sequons and allows their diminishing over time due to genetic drift as mentioned above to form the sequon-poor protein class.

Such complementarity further implies the general stability offered by sequons as an ancient function in protein sequences. Structural and sequence analyses have pinpointed the Asx motifs^22^ and the ST-motifs^23^, which can cap helices and facilitate turns in protein structures. It is also observed that these sequences can switch with each other and still maintain the same structural selection^23^. It is therefore not surprising to find Asx and ST motifs joined together in sequons to facilitate protein folding. In fact, the N-linked sequons being recognized by OST for N-glycosylation is because it can form Asx-turns and β-turns^24–26^, in which a hydrogen bond can be formed between Asn and Ser/Thr. Recently, studies have also indicated that the stability offered by N-glycosylation can be replaced by charged and polar amino acid clusters^27^. These studies agreed with our observation of the enrichment of these amino acids in the sequon-poor sequences.

In contrast to sequon-poor proteins, interestingly, in cyto-proteins, a class of sequon-rich proteins also exists. Their functional enrichment analysis highly focused on DNA and RNA binding, repressors and activators. It is also known that Asn has bidentate nucleic acid binding properties through hydrogen bonding^28^, and it binds preferentially with adenine over guanine^28,29^ in the main groove. In the enrichment analysis, C2H2 zinc finger proteins were highly enriched in this subclass (Fig. 7). The characteristics of the fold of C2H2 zinc finger proteins are a loop/turn flanked by β-sheets and an ɑ-helix joined together by the zinc ion through two Cys on the two β-sheets and the two His from the ɑ-helix^30^. These discoveries prompted us to further investigate these proteins.

**Figure 8:**
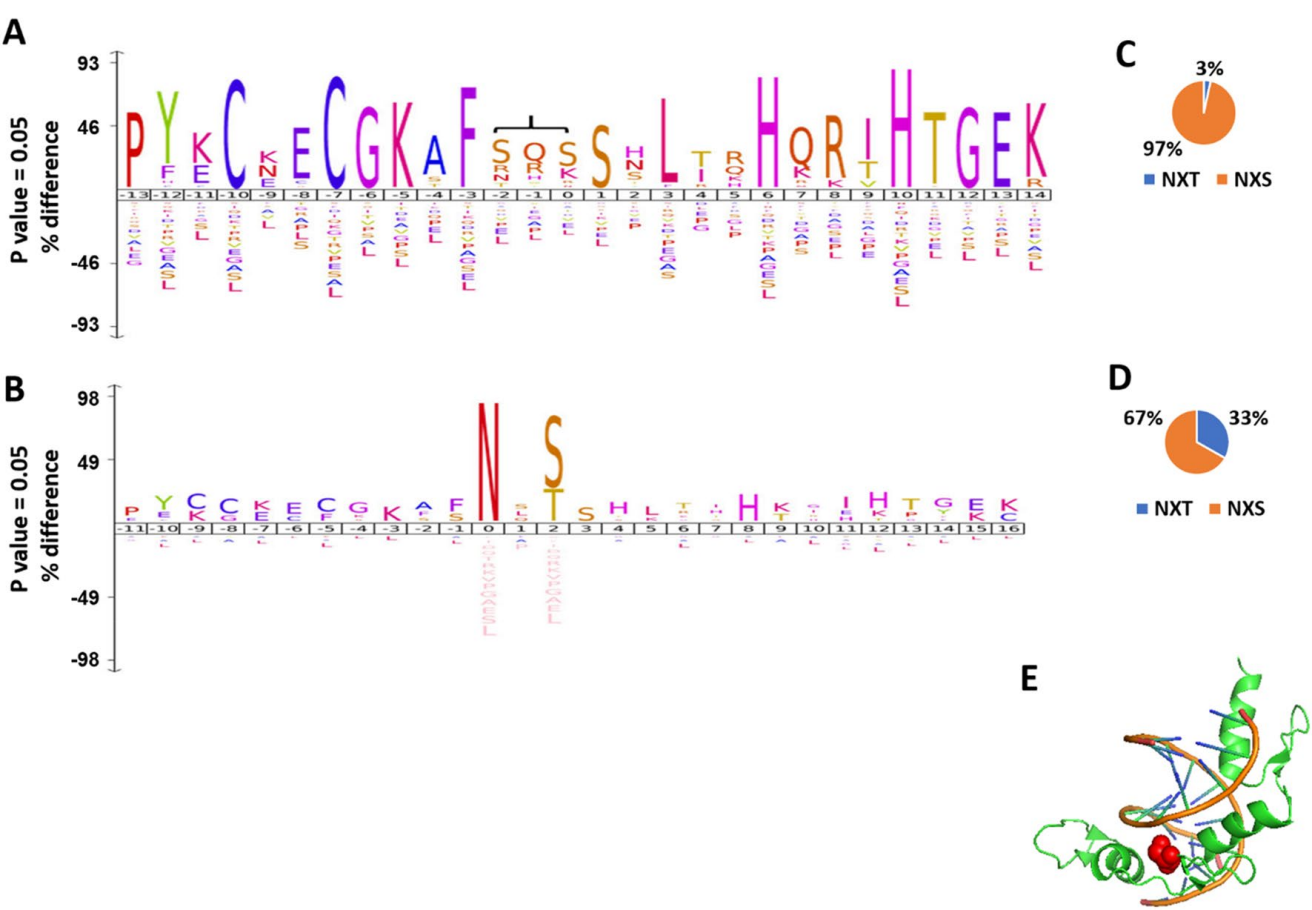
The amino acid sequence pattern in C2H2 zinc finger motifs and in sequons of C2H2 zinc finger proteins. (**A**) The 28 amino acid zinc finger domain enriched in C2H2 zinc finger proteins, and the region where sequon resides is highlighted by the bracket; (**B**) Enriched sequences around sequons in C2H2 zinc finger proteins; (**C**) Distribution of NXT and NXS in sequons of C2H2 zinc finger domains; (**D**) Distribution of NXT and NXS in sequons of C2H2 zinc finger proteins; (**E**) Structure of ZIF268, PDB entry 1AAY. The DNA is labeled in brown, peptide secondary structure is in green, and the side chain of serine 45 in sequon (NFS) is highlighted in red.

To study the potential relationship between the cyto-sequon proteins and zinc finger proteins, we pulled all C2H2 zinc finger proteins from the Interpro database (https://www.ebi.ac.uk/interpro/) using ID: IPR036236. Their zinc finger domains were obtained from UniProt annotation. We used IceLogo^12^ to analyze their amino acid distribution within the extended C2H2 zinc finger domains spanning 28 amino acids, as shown in Fig. 8A. The patterns agreed well with published results^30–32^. We then obtained the sequon locations in all the selected zinc finger proteins regardless of the location of the zinc finger domains and used IceLogo to study the amino acid distribution around the sequons, as shown in Fig. 8B. In Fig. 8A, the NXS sequon was clearly enriched in the center region between the two β-sheets and the ɑ-helix. Interestingly, in Fig. 8B, the C2H2 zinc finger domain sequence is also clearly enriched in the vicinity 28 amino acids of all the sequons. These results suggested that the sequon can be part of the zinc finger domain. A further study also showed that as a polar amino acid, serine shares many characteristics with asparagine in that both can form hydrogen bonds, and serine can also bind DNA even though it is weaker than asparagine^28,33^. Serine preferentially binds the DNA backbone, interacts with guanine and potentially provides DNA sequence selection^28^.

NXS is preferentially enriched over NXT in the zinc finger domains shown in Fig. 8A, which prompted us to further compare it with the distribution of the entire cyto-sequon proteins, as shown in Fig. 3 insert. Using similar pie charts, we showed in Fig. 8C,D the NXS and NXT distribution in sequons of both C2H2 zinc finger domains and C2H2 zinc finger proteins. Clearly, in both cases, the NXS was more enriched than those in the cyto-sequons shown in Fig. 3, and shockingly, in the zinc finger domain, the sequons were almost exclusively NXS. Such a drastic difference clearly suggests that the presence of NXS in zinc finger is under certain selection, and this selection is opposite to N-glycosylation selection in the secreted-sequons and ecto-sequons in TM-sequon proteins, as shown in the insert of Fig. 3.

The position of the zinc-finger sequon, as shown in Fig. 8A, is at the turn portion in the middle of the zinc finger fold, which is the C-terminus of the β-sheet and the N-terminus of the ɑ-helix. This turn structure agrees with the known Asx^22^and ST^23^motifs and is also similar to the fold observed in the sequon and OST complex^24,25^. Further consistency came from the highly enriched charged and polar amino acids at the same position in the middle of the zinc finger domain, if not a sequon, as shown in Fig. 8A, which includes Arg and Lys. These results agreed with the stability that charged and polar amino acids can provide in mimicking and substituting the interaction exerted by a sequon in N-glycoproteins for peptide folding^27^. The uniqueness here in zinc finger domains is that these charged and polar amino acids also have affinity to nucleic acids^29,33,34^.

To further investigate the potential function of sequons in the zinc finger domains, we examined the known crystal structure of the zinc finger domain in complex with its target DNA in the Protein Database Bank (PDB). From the X-ray diffraction structure of the ZIF268 zinc finger-DNA complex, entry 1AAY in PDB, obtained from a 1.6 Å resolution^35^, the sequon (NFS) was in the middle of the second finger of the three-finger peptide. ZIF268 is a well-studied model in zinc finger proteins, and its structure has been resolved from both 2.1 and 1.6 Å^35,36^. These high-resolution detailed structures allowed a better understanding of the nucleic acid binding properties of zinc finger domains, which is part of the actively developing area of genome editing^37,38^. As shown in Fig. 8E, the sequon was located at the edge of the DNA major groove, with serine (Ser 45) forming a hydrogen bond with the phosphate group of the guanine (G 6)^35^. In addition, the DNA conformation was slightly changed to accommodate the binding of three fingers^35^. This tight space and orientation may explain why the position is highly selected for serine instead of threonine.

## Conclusions

In summary, our studies of cyto-sequon proteins in comparison with TM and secreted sequon proteins discovered that sequons are frequently observed in cytoproteins (Fig. 1). Their occurrence is correlated with protein length in general and can be described by a linear model (Figs. 5 and 6), and the longest cytoprotein, Titin, has the highest number of sequons. When sequons are utilized for functions, their density increases, and their spacing decreases. These characteristics can be observed in a subset of TMs and secreted sequons where they are used for N-glycosylation (Figs. 4 and 5) and in nucleic acid binding proteins such as zinc finger proteins (Fig. 8). In these functional motifs, sequons are often located in turns^39^. In addition, the distribution between NXS and NXT deviates from the ratio of S and T in the human proteome when sequons are selected for function. In particular, our studies discovered that ecto-sequons, including secreted-sequon proteins and ecto-sequons in TM-sequon proteins (Fig. 2 and Supplementary Table S1), are prone to NXT (Fig. 3 insert), whereas zinc finger proteins are selective to NXS (Fig. 8C,D). Our study also identified a subset of sequon-poor proteins as outliers that did not follow the length correlation (Supplementary Tables S2 and S3). For sequon-poor cytoproteins, charged and polar amino acids were enriched, and their corresponding PTMs, such as phosphorylation, ubiquitination, and alkylation carried by charged and polar amino acids, were also enriched (Fig. 7). In the case of TM and secreted proteins, the complementary PTMs to N-glycosylation-poor proteins were TM domains (Fig. 7), similar to what we discovered before^13^. We also discovered here that the secreted sequon-poor proteins, disulfide bridges and O-glycosylation were complementary to N-linked sequons (Fig. 7). Finally, for the sequon-rich cyto-proteins (Supplementary Table S4), we discovered the high involvement of sequons in nuclear acid binding (Figs. 7 and 8). We hope these newly discovered trends and roles in sequons can assist better understanding of the structure and function of proteins through their sequences. The new role of sequons in zinc finger domains (Fig. 8) could help to refine different modes of DNA recognition by zinc fingers in genome editing. Collectively, the new layer of information acquired here should contribute to future protein design and engineering.

### Supplementary Information

Supplementary Information.

## Data Availability

All data generated or analysed during this study are included in this published article [and its supplementary information files].
